# Fucoidan Suppresses Mitochondrial Dysfunction and Cell Death against 1-Methyl-4-Phenylpyridinum-Induced Neuronal Cytotoxicity via Regulation of PGC-1α Expression

**DOI:** 10.3390/md17090518

**Published:** 2019-09-02

**Authors:** Yong-Seok Han, Jun Hee Lee, Sang Hun Lee

**Affiliations:** 1 Medical Science Research Institute, Soonchunhyang University Seoul Hospital, Seoul 04401, Korea (Y.-S.H.) (J.H.L.); 2 Departments of Biochemistry, Soonchunhyang University College of Medicine, Cheonan 31151, Korea

**Keywords:** fucoidan, PGC-1α, Parkinson’s disease, mitochondrial dysfunction

## Abstract

Mitochondria are considered to be the powerhouses of cells. They are the most commonly damaged organelles within dopaminergic neurons in patients with Parkinson’s disease (PD). Despite the importance of protecting neuronal mitochondria in PD patients, the detailed mechanisms underlying mitochondrial dysfunction during pathogenesis and pathophysiological progression of PD have not yet been elucidated. We investigated the protective action of fucoidan against the detrimental action of 1-methyl-4-phenyl-pyridinium (MPP^+^), a neurotoxin used to model PD, in the mitochondria of SH-SY5Y neural cells. Fucoidan increased the expression of peroxisome proliferator-activated receptor gamma coactivator-1 alpha (PGC-1α) and protected the cells from MPP^+^-induced apoptosis by upregulating the 5′ adenosine monophosphate-activated protein kinase (AMPK)-PGC-1α axis. These effects were blocked by the silencing of the PGC-1α axis. These results indicated that fucoidan protects SH-SY5Y cells from mitochondrial dysfunction and cell death caused by MPP^+^ treatment, via the AMPK-PGC-1α axis. These findings also suggest that fucoidan could potentially be used as a therapeutic agent for PD.

## 1. Introduction

Parkinson’s disease (PD) is the second most common chronic progressive neurodegenerative disorder. It is characterized by the selective loss of dopaminergic neurons and formation of Lewy bodies in the substantia nigra of the brain [1]. Although the pathogenic mechanisms underlying PD remain to be elucidated, strong evidence suggests that oxidative stress may be involved in the pathogenesis of PD [2]. The association between PD and mitochondrial dysfunction, in particular, indicates that oxidative stress may lead to inhibition of the electron transport chain, excessive generation of reactive oxygen species (ROS), and deletions in mitochondrial DNA [3,4,5,6]. Therefore, the mechanisms underlying the relationship between PD pathogenesis and mitochondrial dysfunction need to be investigated.

Peroxisome proliferator-activated receptor gamma coactivator-1 alpha (PGC-1α) is a pivotal regulator of mitochondrial biogenesis and oxidative metabolism [7]. PGC-1α plays a central role in the mitochondrial regulatory network. Its role includes transcriptional regulation of mitochondrial biogenesis and respiratory function [8]. PGC-1α increases the expression of mitochondrial respiratory chain complexes I, II, III, and IV, and inhibits dopamine neuron loss in a cellular PD model [9]. PGC-1α acts as a key factor responsible for inducing an antioxidant effect against oxidative stress in neurons [10]. In neural cells, PGC-1α regulates several ROS detoxifying enzymes, including superoxide dismutase (SOD) 1, SOD2, glutathione peroxidase-1, and catalase, which inhibit oxidative-stressor-mediated cell death [10]. Thus, several studies have focused on the therapeutic effect of PGC-1α as well as on its role in the pathogenesis of neurodegenerative diseases.

Fucoidan is a marine sulfated polysaccharide, extracted from edible brown algae and seaweeds. Several studies have described its biological activities, including anti-tumor, anti-inflammatory, and anti-oxidant activities [11,12,13]. In addition, fucoidan acts as a stem cell priming agent which enhances the bioactivities and therapeutic effects of stem cells [14,15,16]. In neurodegenerative diseases, fucoidan also protects neurons against treatments with 1-methyl-4-phenyl-1,2,3,6-tetrahydropyridine (MPTP), lipopolysaccharide, and 6-hydroxydopamine, thereby indicating its potential as a therapeutic agent against PD [17,18,19]. However, detailed molecular mechanisms underlying the protective effect of fucoidan on neurodegenerative diseases remain unclear. The objective of the current study was to evaluate the effect of fucoidan on ROS generation and mitochondria function in neuronal cells treated with 1-methyl-4-phenyl-pyridinium (MPP^+^). Furthermore, in this study, we elucidated the mechanisms underlying the protective effect exerted by fucoidan against MPP^+^-induced cell death through the regulation of PGC-1α.

## 2. Results

### 2.1. Protective Effects of Fucoidan against MPP^+^-Induced Cytotoxicity

**Figure 1:**
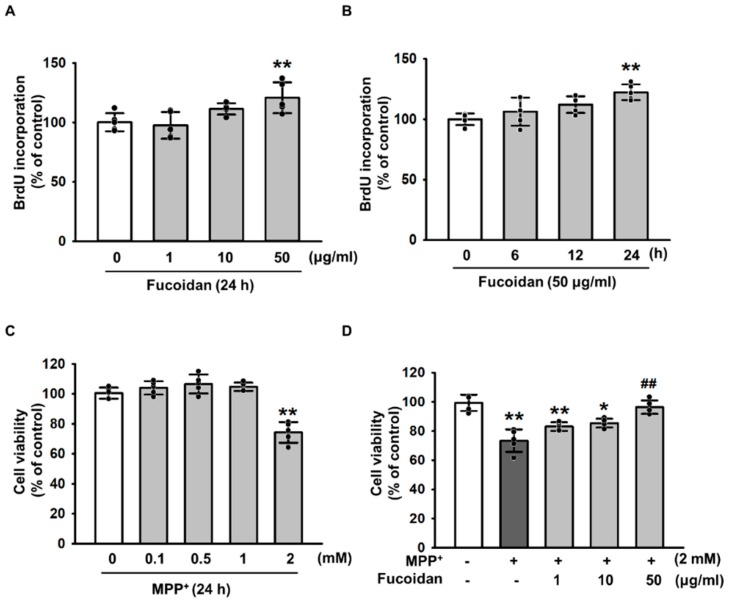
Effect of fucoidan on MPP^+^-induced apoptosis. (**A**) SH-SY5Y cells were exposed to 0–50 μg/mL fucoidan for 24 h, and 5-bromo-2′-deoxyuridine (BrdU) incorporation was quantified by measuring the absorbance at 690 nm. Values represent the mean ± SD (*n* = 5). * *p* < 0.05 and ** *p* < 0.01 vs. control. (**B**) SH-SY5Y cells were exposed to 50 μg/mL fucoidan for 0–24 h, and BrdU incorporation was quantified by measuring the absorbance at 690 nm. Values represent the mean ± SD (*n* = 5). * *p* < 0.05 and ** *p* < 0.01 vs. control. (**C**) SH-SY5Y cells were exposed to 0–2 mM MPP^+^ for 24 h, and BrdU incorporation was measured by absorbance detection. Values represent the mean ± SD (*n* = 5). ** *p* < 0.01 vs. control. (**D**) SH-SY5Y cells were treated with 2 mM MPP^+^ for 24 h before pretreatment of the cells with fucoidan (1, 10 and 50 μg/mL, for 24 h). Values represent the mean ± SD (*n* = 5). * *p* < 0.05 and ** *p* < 0.01 vs. control, ## *p* < 0.01 vs. MPP^+^ only.

To investigate the protective effects of fucoidan against oxidative stress–induced cytotoxicity by MPP^+^, we first treated SH-SY5Y cells with various concentrations of fucoidan. As a result, fucoidan stimulated cell proliferation at 24 h with 50 μg/mL (Figure 1A,B). In addition, SH-SY5Y were treated with MPP^+^ for the indicated dose periods (0, 0.1, 0.5, 1, and 2 mM). Maximum MPP^+^-induced decrease in SH-SY5Y viability was observed at 2 mM (Figure 1C). However, the decrease in viability from MPP^+^-induced apoptosis was prevented by pretreatment with fucoidan (Figure 1D). To further investigate the protective effect of fucoidan against MPP^+^-induced senescence in SH-SY5Y cells, the levels of SMP30 were quantified by western blotting and senescence-associated β-galactosidase assay were performed. However, these results showed that MPP^+^ did not induce senescence in SH-SY5Y cells (Supplemental Figure S1). These results indicate that fucoidan protects SH-SY5Y cells from apoptosis induced by MPP^+^. These findings suggest that fucoidan increases the proliferation of SH-SY5Y cells and cell viability in a model of MPP^+^-induced PD.

### 2.2. Fucoidan-Mediated Inhibition of MPP^+^-Induced Oxidative Stress and Mitochondrial Dysfunction

**Figure 2:**
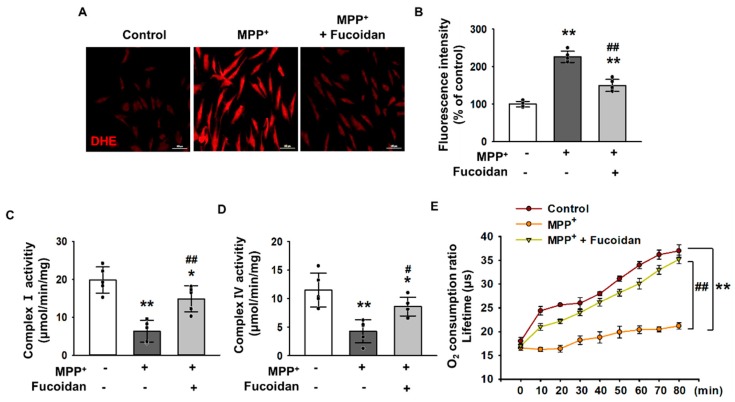
The protective effect of fucoidan on MPP^+^-induced oxidative stress and mitochondrial dysfunction. (**A**) SH-SY5Y cells were treated with 2 mM MPP^+^ before fucoidan (50 μg/mL, for 24 h) pretreatment. Representative images of DHE fluorescence staining that indicate generated ROS amounts in different treatment groups. Scale bar = 100 μm. (**B**) The quantification of DHE fluorescence levels. The values represent the means ± SD. ** *p* < 0.01 vs. control, ## *p* < 0.01 vs. MPP^+^. (**C**,**D**) Complex I and IV enzyme activity assay in MPP^+^ (2 mM, for 24 h)-exposed SH-SY5Y cells pretreated with fucoidan (50 μg/mL, for 24 h) (*n* = 5). The values represent the means ± SD. * *p* < 0.05 and ** *p* < 0.01 vs. control, # *p* < 0.05 and ## *p* < 0.01 vs. MPP^+^. (**E**) Oxygen consumption ratio in SH-SY5Y cells, SH-SY5Y cells + MPP^+^ and SH-SY5Y cells + MPP^+^ (2 mM, for 24 h) + fucoidan (50 μg/mL, for 24 h) (*n* = 5). The values represent the means ± SD. ** *p* < 0.01 vs. control, ## *p* < 0.01 vs. MPP^+^.

To examine the effects of fucoidan on oxidative stress in response to MPP^+^ treatment, MPP^+^-treated cells were incubated with dihydroethidium (DHE) for ROS detection, and changes in oxidative stress levels were determined using fluorescent microscopic imaging. The levels of oxidative stress were significantly higher in SH-SY5Y cells exposed to 2 mM MPP^+^ for 24 h compared to that in untreated cells, indicating that MPP^+^ enhances oxidative stress (Figure 2A,B). Fluorescent microscopic imaging for DHE revealed that treatment with fucoidan significantly attenuated the increase in oxidative stress levels (Figure 2A,B). Furthermore, to determine whether MPP^+^ reduced mitochondrial membrane potential through oxidative stress, complex I & IV activities (Figure 2C,D) and the mitochondrial O_2_ consumption ratio (Figure 2E) were measured. The results indicate that MPP^+^ decreased complex I & IV activities and mitochondrial O_2_ consumption ratio. In contrast, fucoidan pretreatment protected complex I & IV activities and the mitochondrial O_2_ consumption ratio from MPP^+^ (Figure 2C–E). However, fucoidan did not alter the oxygen consumption ratio in SH-SY5Y cells in the absence of MPP^+^ (Supplemental Figure S2), suggesting that it may affect mitochondrial oxidative phosphorylation under conditions of MPP^+^-induced stress. These results indicate that fucoidan protects SH-SY5Y cells from MPP^+^-induced oxidative stress and mitochondrial dysfunction.

### 2.3. Fucoidan Enhanced PGC-1α Expression in SH-SY5Y via Regulation of Phosphorylation-AMPK

**Figure 3:**
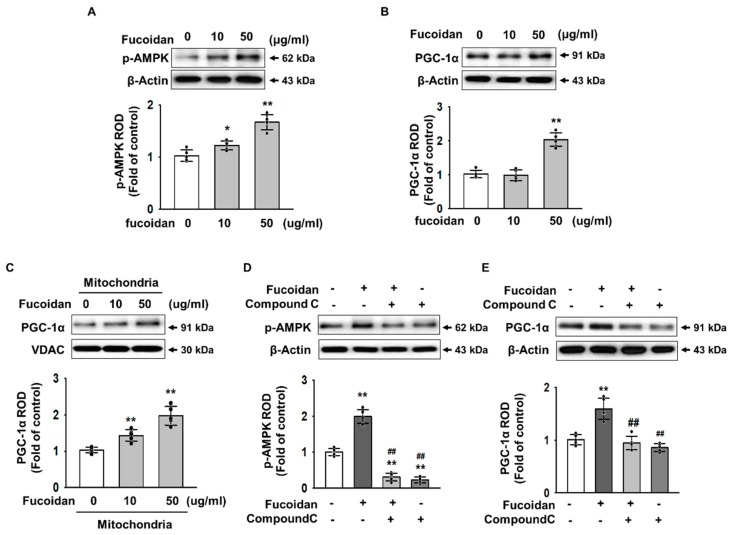
Fucoidan regulates the expression of PGC-1α via p-AMPK. (**A**,**B**) Western blotting for p-AMPK and PGC-1α in fucoidan (0–50 μg/mL, for 24 h)-treated SH-SY5Y cells (*n* = 5). The values represent the means ± SD. * *p* < 0.05 and ** *p* < 0.01 vs. control. (**C**) Western blotting for PGC-1α in mitochondria of fucoidan (0–50 μg/mL, for 24 h)-treated SH-SY5Y cells (*n* = 5). The values represent the means ± SD. ** *p* < 0.01 vs. control. (**D**,**E**) Western blotting for p-AMPK and PGC-1α in fucoidan (50 μg/mL, for 24 h)-treated SH-SY5Y cells pretreated with compound C (AMPK inhibitor, 1 μM, for 30 min) (*n* = 5). The values represent the means ± SD. ** *p* < 0.01 vs. control, ## *p* < 0.01 vs. MPP^+^.

To investigate the key mediator of fucoidan-protected mitochondria function, we analyzed 5′ adenosine monophosphate-activated protein kinase (AMPK) and PGC-1α expression using western blot analysis in a dose-dependent manner (0–50 μg/mL). Western blot analysis showed that phosphorylation of AMPK was increased in SH-SY5Y treated with fucoidan (50 μg/mL) (Figure 3A). PGC-1α was also found to increase expression at the same concentration (Figure 3B). In addition, PGC-1α increased influx into the mitochondria (Figure 3C). Furthermore, we utilized the AMPK inhibitor Compound C to confirm that fucoidan activates PGC-1α by regulating AMPK phosphorylation. As a result, when the phosphorylation of AMPK was inhibited, fucoidan did not increase the expression of PGC-1α in SH-SY5Y cells (Figure 3D,E). These results indicate that fucoidan regulates the expression of PGC-1α, a major factor of mitochondrial biogenesis via phosphorylation of AMPK.

### 2.4. Fucoidan Protected Mitochondria Function in SH-SY5Y via Regulation of PGC-1α Expression

**Figure 4:**
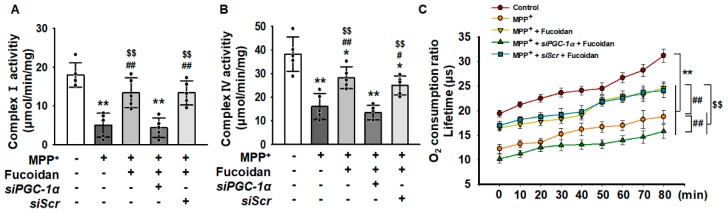
Fucoidan protects mitochondrial function by regulating PGC-1α expression. (**A**,**B**) Complex I and IV enzyme activity assay in fucoidan (50 μg/mL, for 24 h)-pretreated SH-SY5Y cells transfected with *siPGC-1α* or *siScr* after treatment with MPP^+^ (*n* = 5). The values represent the means ± SD. * *p* < 0.05 and ** *p* < 0.01 vs. control, # *p* < 0.05 and ## *p* < 0.01 vs. MPP^+^, $$ *p* < 0.01 vs. fucoidan + MPP^+^. (**C**) Oxygen consumption ratio in fucoidan (50 μg/mL, for 24 h)-pretreated SH-SY5Y cells transfected with *siPGC-1α* or *siScr* after treatment with MPP^+^ (*n* = 5). The values represent the means ± SD. ** *p* < 0.01 vs. control, ## *p* < 0.01 vs. MPP^+^, $$ *p* < 0.01 vs. MPP^+^ + fucoidan.

To clarify whether the reduced mitochondrial membrane potential was caused by a decreased expression of PGC-1α, we measured complex I & IV activities and mitochondrial O_2_ consumption ratio. As a result, when the expression of PGC-1α was inhibited, the fucoidan did not increase the activities of complex I & IV (Figure 4A,B), and the O_2_ consumption ratio of the mitochondria was also inhibited by inhibiting the expression of PGC-1α (Figure 4C). These results indicated that the effect of fucoidan reduced dysfunctional mitochondria by increasing PGC-1α expression.

### 2.5. Fucoidan Protection against MPP^+^-Induced Apoptosis via Regulation of PGC-1α Expression

**Figure 5:**
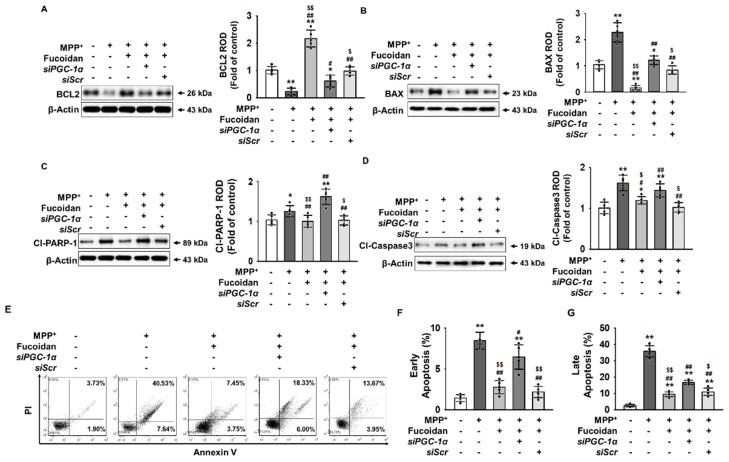
Fucoidan protects SH-SY5Y cells from MPP^+^-induced apoptosis by regulating PGC-1α expression. (**A**–**D**) Western blotting for the quantification of BCL2, BAX, Cl-PARP-1, and Cl-Caspase3 in fucoidan (50 μg/mL, for 24 h)-pretreated SH-SY5Y cells transfected with *siPGC-1α* or *siScr* after treatment with MPP^+^ (*n* = 5). The values represent the means ± SD. * *p* < 0.05 and ** *p* < 0.01 vs. control, # *p* < 0.05 and ## *p* < 0.01 vs. MPP^+^, $ *p* < 0.05 and $$ *p* < 0.01 vs. fucoidan + MPP^+^. (**E**) Flow cytometry analysis for analyzing the PI and Annexin V staining of fucoidan (50 μg/mL, for 24 h)-pretreated SH-SY5Y cells transfected with *siPGC-1α* or *siScr* after treatment with MPP^+^. (**F**) Early apoptosis was quantified as the percentage of Annexin V-positive and PI-negative cells (*n* = 5). Values represent the mean ± SD. ** *p* < 0.01 vs. control, # *p* < 0.05 and ## *p* < 0.01 vs. MPP^+^, $$ *p* < 0.01 vs. fucoidan + MPP^+^. (**G**) Late apoptosis was quantified as the percentage of Annexin V-positive and PI-positive cells (*n* = 5). Values represent the mean ± SD. ** *p* < 0.01 vs. control, ## *p* < 0.01 vs. MPP^+^, $ *p* < 0.05 and $$ *p* < 0.01 vs. fucoidan + MPP^+^.

To explore the effects of fucoidan on the induction of apoptosis by MPP^+^, western blot assay was used to analyze the expression levels of B-cell lymphoma 2 (BCL2), BCL-2-like protein 4 (Bax), cleaved-poly (ADP-ribose) polymerase 1 (PARP-1), and cleaved-caspase-3, which are associated with cell survival or apoptosis. When apoptosis was induced in SH-SY5Y cells through oxidative stress caused by MPP^+^, fucoidan increased BCL2 expression, while the expression levels of Bax, cleaved-PARP-1, and cleaved-caspase-3 were significantly lower (Figure 5A–D). In addition, apoptosis was evaluated using propidium iodide (PI)/annexin V staining. As a result, fucoidan reduced apoptosis via PGC-1α expression regulation (Figure 5E–G). These results indicate that the effect of fucoidan inhibits apoptosis by increasing PGC-1α expression.

## 3. Discussion

Fucoidan has been reported to exert various biological effects, such as anti-tumor, immunomodulatory, anti-viral, anti-thrombotic, anti-coagulant, anti-inflammatory, and anti-oxidant effects [12,13,20,21,22,23,24]. Recent studies have indicated the non-classical activities of fucoidan, including the inhibition of VEGF secretion under oxidative stress conditions in ocular cells, alleviation of metabolic syndrome, protection of the gastrointestinal tract, regulation of angiogenesis, contribution to bone growth and health, and prevention of hippocampal neuronal cell death from cerebral ischemia [25,26,27,28]. Furthermore, previous studies conducted by us have revealed that fucoidan shows protective effects against cellular senescence in endothelial colony-forming cells [14]. Fucoidan also protects mesenchymal stem cells (MSCs) from oxidative stress-induced cell death through regulation of MnSOD activity [15]. In addition, fucoidan enhances bioactivities of MSCs under chronic kidney disease condition [16,29]. In PD, fucoidan has been shown to exert therapeutic effects on MPTP and rotenone-induced neurotoxicity in dopaminergic neurons [18,30]. Although the therapeutic effect of fucoidan on several chronic diseases is evident, the mechanisms underlying its neuroprotective effect remain unclear. In this study, we investigated the protective effects of fucoidan in PD. The results indicated that fucoidan inhibits MPP^+^-induced generation of ROS, dysfunction of mitochondrial oxidative phosphorylation, and cellular apoptosis in SH-SY5Y cells through regulation of AMPK-PGC-1α axis.

The beneficial activities of fucoidan are related to its chemical structure. The structure of native fucoidan from brown seaweeds is usually heterogeneous and branched. In particular, several sugar monomers such as glucose, galactose, mannose, and xylose and many different sulfation patterns are linked to the unique structure of fucoidan, including its monomeric composition, molecular weight, sulfate content, and sulfation pattern [31]. These different structures of fucoidan isolated from various brown seaweeds and algae have different biological activities [32]. Although the biological activities of crude and purified fucoidan isolated from *Sargassum cristaefolium* differ, but not to a very large extent, the extraction yield and efficiency of bioactivities are different [33]. Molecular weight is a pivotal factor affecting fucoidan activity. Furthermore, the relationship between fucoidan and its bioactivity is not simply linear. Low molecular weight fucoidan (under 10 kDa) derived from *Undaria pinnatifida* has been shown to significantly exhibit greater anti-cancer effects than that purchased from Sigma-Aldrich [34]. Sulfated and benoylated fucoidan isolated from *Saccharina japonica* showed better neuroprotective activity than low molecular weight fucoidan [35]. The bioactivities of fucoidan from *Fucus vesiculosus* and 18 gradually depolymerized fractions decreased with decreasing molecular weight [36]. Despite intensive studies on fucoidan, the high variability of substance is due to its structural features, such as its source, molecular weight, percentage of sulfate groups. In this study, we used crude fucoidan from *Fucus vesiculosus* purchased from Sigma-Aldrich Chemical, which has a weight average molecular mass of 38.2 kDa as determined by size exclusion chromatograph [37]. The backbone of this commercial crude fucoidan is a polymer of alternating (1→3)-linked α-l-fucopyranose and (1→4)-linked α-l-fucopyranose residues and the presence of sulfate groups on both *O*-2 and *O*-3 [38,39,40]. This crude fucoidan is composed of fucose (44.1%), sulfate (26.3%), ash (31.1%), and a little aminoglucose [41,42]. Crude fucoidan from *Fucus vesiculosus* inhibits angiogenesis in osteosarcoma [43]. Furthermore, our previous studies have shown that treatment of endothelial colony forming cells and mesenchymal stem cells with this type of fucoidan improves neovascularization in ischemic diseases [14,15]. These results indicate that the activity of fucoidan differs depending on cell type. Most previous studies have only analyzed one type of fucoidan, suggesting that each type of fucoidan isolated from various sources needs to be investigated in a target tissue- and cell-dependent manner.

Fucoidan induces the proliferation of several types of cells. In endothelial colony forming cells, fucoidan improves proliferation of senescent cells through phosphorylation of focal adhesion kinase (FAK)-extracellular signal-regulated kinase (ERK) signaling cascade [14]. Fucoidan shows high affinity for integrin αMβ2, which mediates the FAK signaling cascade [44,45]. Activation of ERK, a molecule downstream of FAK, enhances cell proliferation [46]. Thus, fucoidan-induced phosphorylation of ERK increases the proliferation of endothelial colony forming cells by enhancing the expression of cyclin E, cyclin D, cyclin-dependent kinases 2 (Cdk2), and Cdk4 [14]. In addition, fucoidan augments MSC proliferation via the FAK-Akt signaling axis [16]. These findings indicate that the interaction between fucoidan and integrin regulates cell proliferation through activation of FAK. The results of our study have shown that fucoidan increased the proliferation of SH-SY5Y cells. Other studies have demonstrated the protective effect of fucoidan on retinal pigment epithelium via the integrin-FAK-PGC-1α pathway [47]. These findings suggest that the effect of fucoidan on proliferation in SH-SY5Y cells could be mediated via FAK-PGC-1α signaling. However, the underlying mechanism by which fucoidan regulates proliferation by regulating FAK-PGC-1α signaling needs further investigation.

To replicate conditions of oxidative degeneration of neurons in the brains of PD patients, classic PD cell models are produced by utilizing MPP^+^, rotenone, or 6-hydroxydopamine as neurotoxins to induce varying degrees of oxidative stress injury [48]. MPTP, which is a neurotoxin, is produced in primates and rodents with PD, and its active metabolite MPP^+^ has been extensively used for the generation of PD animal models [49,50]. Thus, MPTP is one of the most well studied biomarkers of PD; it inhibits the activity of mitochondrial complex I in the form of MPP^+^, causing the disruption of electron flow and overproduction of ROS [51]. In the MPP^+^-induced PD cell model, MPP^+^ inactivates tyrosine hydroxylase in dopaminergic neurons, thereby inhibiting oxidative stress–induced injury of mitochondrial complex I [52]. This leads to superoxide (O_2_ 2•) generation and ATP depletion [53,54,55]. Although O_2_2• itself is not detrimental, it can react rapidly with nitric oxide to form peroxynitrite, which in turn decomposes rapidly to form cell-damaging hydroxyl radicals under physiological pH conditions [56,57], suggesting that MPP^+^ induced generation of oxygen free radicals within mitochondria are responsible for PD pathogenesis. Furthermore, recent studies have indicated that MPP^+^ may induce the opening of mitochondrial permeability transition pores, causing the disruption of mitochondrial membrane potential, impairment of ATP production, and generation of ROS, which ultimately induces dopaminergic neuron apoptosis [58,59]. Involvement of mitochondrial integrity in the degenerative processes of PD in patients remains a subject for future research [60]. Results of the current study indicated that MPP^+^ treatment reduced the viability of SH-SY5Y cells in a dose-dependent manner, whereas pretreatment with fucoidan protected the cells from MPP^+^-induced apoptosis by mitigating oxidative stress and enhancing the activities of mitochondrial complexes I and IV. In particular, fucoidan did not alter the mitochondrial oxidative phosphorylation in SH-SY5Y cells under normal conditions (in cells not subjected to MPP^+^ treatment). Energy homeostasis is a key determinant of survival in all organisms. Under physiological conditions, cells consistently maintain energy metabolism homeostasis by regulating glycolytic flux, mitochondrial oxidative phosphorylation, and metabolic-associated cell signaling, depending on the availability of nutrients and oxygen [61,62]. Some pathophysiological conditions, such as hypoxia, imbalance of ROS levels, limitation of nutrients, and direct damage to mitochondria, induce mitochondrial metabolic alterations, such as switching of mitochondrial oxidative phosphorylation to glycolysis and fatty acid-mediated oxidative phosphorylation [61,62,63,64]. Owing to the maintenance of mitochondrial homeostasis under physiological conditions, fucoidan might not alter mitochondrial oxidative phosphorylation in SH-SY5Y cells. In line with our results, several studies also have shown that fucoidan does not affect mitochondrial function in dopaminergic neurons and mouse brain tissue under physiological conditions [30,65]. However, the mechanism underlying the effect of fucoidan on mitochondrial function under physiological conditions remains unclear. These findings suggest that fucoidan exerts a direct neuroprotective effect on MPP^+^-induced neuronal injury by protecting mitochondrial oxidative phosphorylation.

PGC-1α is a transcriptional co-activator that plays a significant role in promoting mitochondrial biogenesis and enhancing mitochondrial function [66,67,68]. A recent meta-analysis reported a decrease in PGC-1α and its downstream genes in postmortem brains of PD patients [9]. Further evidence suggests that the expression of genes involved in mitochondrial respiration was markedly decreased, dopaminergic neurons were more sensitive to MPTP in PGC-1α knockout mice [69,70], and PGC-1α overexpression increased the expression of nuclear-encoded subunits of the mitochondrial respiratory chain and prevented dopaminergic neuronal degeneration in models with mutant α-synuclein, rotenone, or MPTP [9,70]. PGC-1α is also shown to enhance mitochondrial capacity for oxidative phosphorylation and trigger the coordinate expression of nuclear-encoded genes driving mitochondrial biogenesis [71]. Our results demonstrated that fucoidan induced PGC-1α expression and that its protective effect, which results in the inhibition of MPP^+^-mediated apoptosis through the prevention of mitochondrial dysfunction, is dependent on PGC-1α expression. The oxygen consumption ratio and activities of complexes I and IV were significantly decreased in the presence of MPP^+^. However, treatment with fucoidan against MPP^+^ treatment increased mitochondrial oxidative phosphorylation significantly, whereas silencing of PGC-1α blocked this effect. Collectively, these data suggest that the protective effect of fucoidan on MPP^+^-induced mitochondrial dysfunction is regulated by PGC-1α.

Our results demonstrated that fucoidan regulated the expression of PGC-1α through the phosphorylation of AMPK. Previous studies show that the consequences of AMPK activation include the acute modulation of metabolism as a result of the phosphorylation of downstream metabolic enzymes and chronic changes in gene expression and mitochondrial biogenesis [72,73]. Effects of AMPK on mitochondrial genes and PGC-1α are almost entirely dependent on the function of PGC-1α protein, and it is effective in promoting mitochondrial biogenesis in various somatic cells [73,74]. Our results further demonstrate that fucoidan employs protective effects via the AMPK-mediated regulation of PGC-1α expression. We found that SH-SY5Y cells lose all protective effects from fucoidan when PGC-1α expression was inhibited; the activities of Complex I and IV were not enhanced even after pretreatment with fucoidan. These findings suggested that fucoidan increased the expression of PGC-1α via phosphorylation of AMPK.

**Figure 6:**
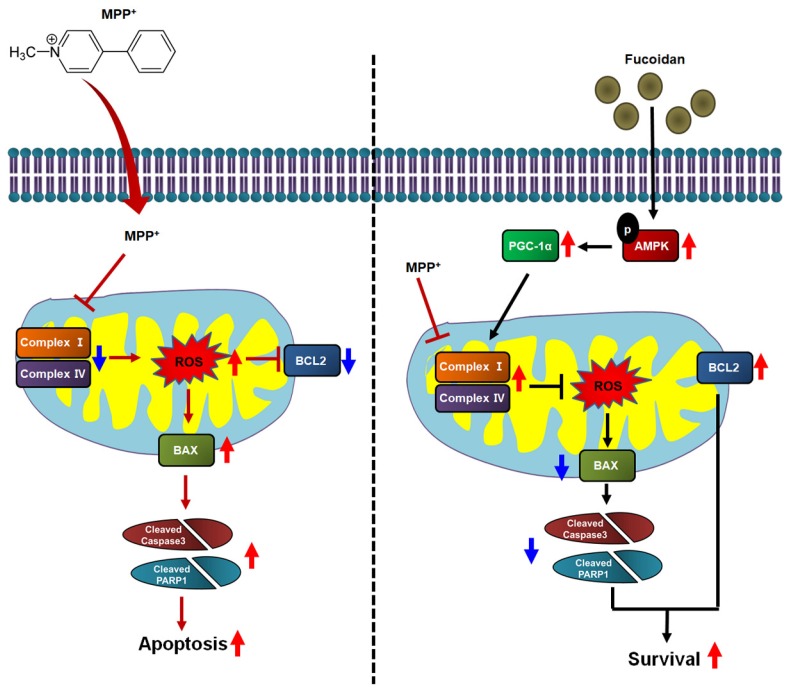
Schematic illustrating the mechanism of fucoidan inhibition of neuronal cytotoxicity via PGC-1α expression regulation. MPP^+^ is a neurotoxic substance that increases ROS by reducing the activities of Complex I and IV in SH-SY5Y cells. In addition, increased intracellular ROS induces mitochondrial dysfunction and eventually leads to apoptosis. However, pretreatment with fucoidan increases the phosphorylation of AMPK and upregulates the expression of PGC-1α in neuronal cells. In addition, PGC-1α expression increases the Complex I and IV activities in mitochondria and inhibits ROS production in SH-SY5Y cells. Therefore, fucoidan exhibits inhibitory effects on neurotoxicity as a result of the regulation of PGC-1α and restoration of mitochondrial function.

The present study revealed that fucoidan showed protective effects on mitochondrial oxidative phosphorylation, including the activities of mitochondrial complexes I and IV and the mitochondrial oxygen consumption rates, against MPP^+^ treatment by increasing AMPK-PGC-1α signaling. Our results also indicated that upregulation of AMPK phosphorylation and PGC-1α expression by fucoidan resulted in a marked increase in mitochondrial function (Figure 6). This increase in mitochondrial function protected against MPP^+^-induced cellular apoptosis, specifically through PGC-1α protein dependent pathways. Fucoidan did not inhibit the expression of MPP^+^-induced pro-apoptosis-related proteins, but enhanced PGC-1α expression and suppressed the PGC-1α-dependent pro-apoptosis signaling pathway, leading to the augmentation of cell survival.

In conclusion, our study revealed that by regulating the AMPK-PGC-1α pathway, crude fucoidan isolated from *Fucus vesiculosus* protects against mitochondrial dysfunction and apoptosis in a PD cell model exhibiting MPP^+^-induced mitochondrial dysfunction, high oxidative stress, and decreased survival rate. These findings suggest that fucoidan may be used to enhance the efficacy of therapeutic strategies against neurotoxicity insults in PD. To determine the precise effect of fucoidan from *Fucus vesiculosus* on the pathogenesis of PD, it is necessary to conduct further studies according to different molecular weights and different amounts of sulfate components in fucoidan.

## 4. Materials and Methods

### 4.1. Cell Culture

Human neuroblastoma SH-SY5Y cells were purchased from the American Type Culture Collection (ATCC, Manassas, VA, USA) and grown in Dulbecco’s modified Eagle’s medium (DMEM)/F12 medium (Gibco, Waltham, MA, USA) containing 10% fetal bovine serum (Gibco), 1% Glutamax (Gibco), 50 U/mL streptomycin, 50 U/mL penicillin G, at 37 °C in humidified incubator (Thermo Fisher Scientific, Wilmington, MA, USA) supplied with 5% CO_2_.

### 4.2. Preparation of Fucoidan

The crude type fucoidan from *Fucus vesiculosus* was purchased from Sigma Aldrich (St. Louis, MO, USA; Cat# F5631; Batch No. SLBP3196V). Several studies have characterized previously that this crude fucoidan from *Fucus vesiculosus* has a weight average molecular mass of 38.2 kDa as determined by size exclusion chromatography using a PL-GPC 50 Plus system with online multi-angle laser light scattering and refractive index detection [37], and its backbone is a polymer of alternating (1→3)-linked α-l-fucopyranose and (1→4)-linked α-l-fucopyranose residues and the presence of sulfate groups on both O-2 and O-3 as determined by definitive methods of carbohydrate structural analysis using QAE-Sepharose column chromatography and GC/MS data of methylation [38,39,40]. This crude fucoidan from *Fucus vesiculosus* is composed of fucose (44.1%), sulfate (26.3%), ash (31.1%), and a little aminoglucose as determined by anion-exchange chromatography [41,42]. The crude fucoidan is dissolved in phosphate-buffered saline (PBS), filter-sterilized using a 0.45-μm pore filter (Sartorius Biotech GmbH, Gottingen, Germany), and stored as fucoidan extracts (20 mg/mL) at 4 °C until used.

### 4.3. Cell Proliferation and Cell Viability Assays

Cell proliferation was examined using the 5-bromo-2′-deoxyuridine (BrdU) incorporation assay. SH-SY5Y cells were cultured in 96-well culture plates (3000 cells/well). SH-SY5Y cells were exposed to fucoidan (0, 1, 10, 50, and 100 μg/mL; crude type isolated from *Fucus vesiculosus*; Sigma-Aldrich, St. Louis, MO, USA; Cat# F5631) for a fixed period of 24 h or to 50 μg/mL fucoidan for 6, 12, 24, or 48 h. BrdU incorporation into the newly synthesized DNA of proliferating cells was assessed using an ELISA colorimetric kit (Roche, Basel, Switzerland), following the manufacturer’s instructions. Cell viability was determined using a modified 3-(4,5-dimethylthiazol-2-yl)-2,5-diphenyltetrazolium bromide (MTT; Sigma-Aldrich) assay, which is based on the conversion of tetrazolium salt 3-(4,5-dimethylthiazol-2-yl)-5-(3-carboxymethoxyphenyl)-2-(4-sulfophenyl)-2-tetrazolium to formazan by mitochondrial dehydrogenase. Formazan was quantified by measuring the absorbance of the samples at 570 nm using a microplate reader (BMG Labtech, Ortenberg, Germany).

### 4.4. Dihydroethidium Staining

To measure superoxide anion levels in cultured SH-SY5Y, the cells were incubated with 10 μM DHE (Sigma-Aldrich) for 30 min at 37 °C. After washing with PBS three times, samples were visualized by fluorescence microscopy (Zeiss, Oberkochen, Germany).

### 4.5. Complex I & IV Activities Assays

The cells were lysed with RIPA lysis buffer (Thermo Fisher Scientific). The activities of complex I & IV assays were performed by means of the complex I & IV assays (Abcam, Cambridge, UK). Of total cell lysates, 100 μg of protein was subjected to these experiments. The activation of complex I & IV assays was quantified by measuring absorbance at 450 nm on a microplate reader (BMG). Proteins were extracted by adding RIPA lysis buffer onto the cells and vortexing, followed by incubation in ice for 1 h. The extracts were then centrifuged at 13,000 rpm for 30 min at 4 °C. The supernatants were harvested into 1mL microtubes. The number of the protein extracts was quantitated using bicinchoninic acid (BCA) assay kit (Thermo Fisher Scientific). First, the BCA reagent solution was put into a flat 96-well microplate with the volume of 100 μL per well, followed by addition of the harvested cell lysate with the volume of 3 μL per well. Then, the plate was incubated at 37 °C for 30 min, and the cell protein quantities were measured under the microplate reader at the wavelength of 562 nm. The standard curve was determined by bovine serum albumin.

### 4.6. Measurement of Extracellular Oxygen Consumption Rate

We measured the extracellular oxygen consumption rate of cells using the Extracellular O_2_ Consumption Assay Kit (cat # ab197243; Abcam). SH-SY5Y cells (5 × 10^3^ cells/well in 150 µL culture media) were plated on 96-well cell culture plates and incubated overnight in a CO_2_ incubator at 37 °C. The culture medium was removed and replaced with 150 μL of fresh culture medium that was supplemented with fucoidan (50 μg/mL) and incubated in a CO_2_ incubator at 37 °C for 30 min. MPP^+^ (2 mM) was added, and the product was incubated in a CO_2_ incubator at 37 °C for 24 h. Then, 10 μL of Extracellular O_2_ Consumption reagent and two drops of prewarmed high-sensitivity mineral oil were added to each well, which were measured using a multidetection microplate reader (Victor3; Perkin Elmer, Billerica, MA, USA). Extracellular O_2_ Consumption reagent was measured at 37 °C for 80 min at 2 min intervals using wavelengths of 340 and 650 nm.

### 4.7. Western Blot Analysis

The whole-cell lysates from SH-SY5Y cells (30 μg protein) were separated by sodium dodecyl sulfate-polyacrylamide gel electrophoresis (SDS-PAGE) in an 8–12% gel, and the proteins were transferred to a nitrocellulose membrane. After the blots were washed with TBST (10 mM Tris-HCl [pH 7.6], 150 mM NaCl, 0.05% Tween 20), the membranes were blocked with 5% skim milk for 1 h at room temperature and incubated with the appropriate primary antibodies: against p-AMPK, PGC-1α, BCL2, BAX, cleaved-caspase-3, cleaved-PARP-1, and β-actin (Santa Cruz Biotechnology, Dallas, TX, USA). The membranes were then washed, and the primary antibodies were detected using HRP-conjugated secondary goat anti-rabbit IgG or goat anti-mouse IgG antibodies (Santa Cruz Biotechnology). The bands were detected by enhanced chemiluminescence (Amersham Pharmacia Biotech, Little Chalfont, UK).

### 4.8. PI/Annexin V Analysis

To determine the level of apoptosis, SH-SY5Y cells were stained with PI and annexin V-FITC kit (Thermo Fisher Scientific) and evaluated using a Cyflow Cube 8 kit (Partec, Münster, Germany). Data were analyzed using standard FSC Express software (De Novo Software, Los Angeles, CA, USA).

### 4.9. Statistical Analysis

All data are expressed as the mean ±standard deviation (SD). Results of all experiments were subject to a one-way analysis of variance (ANOVA). Comparisons of more than three groups were performed using the Tukey’s post hoc test. Differences were considered to be statistically significant if *p* < 0.05.

## Supplementary Materials

The following are available online at https://www.mdpi.com/1660-3397/17/9/518/s1. Figure S1. Effect of fucoidan and MPP^+^ on senescence in SH-SY5Y cells. (**A**) Senescence-associated β-galactosidase (β-gal) staining in SH-SY5Y cells treated with fucoidan and/or MPP^+^. Scale bar = 200 μm. (**B**) Western blotting analysis for quantifying senescence marker protein 30 (SMP30) in SH-SY5Y cells treated with fucoidan and/or MPP^+^ (*n* = 3). The values represent the means ± SDs. n.s = no significance. Figure S2. Effect of fucoidan on mitochondrial oxygen consumption in SH-SY5Y cells. Mitochondrial oxygen consumption ratio in SH-SY5Y cells after treatment with fucoidan (50 μg/mL) for 24 h (*n* = 5). The values represent the means ± SDs. n.s = no significance.
