# Supplementary material for: Fucoidan Suppresses Mitochondrial Dysfunction and Cell Death against 1-Methyl-4-Phenylpyridinum-Induced Neuronal Cytotoxicity via Regulation of PGC-1α Expression
Source: Mar Drugs. 2019 Sep 2;17(9):518. doi: 10.3390/md17090518 (PMC6780744; doi:10.3390/md17090518)
Supplement: Supplementary file 1 [file marinedrugs-17-00518-s001.pdf]

Supplemental Materials and Methods

Senescence-associated beta-galactosidase (SA-β-gal) activity

The cells were washed twice with phosphate-buffered saline (PBS) and fixed with 2% formaldehyde/0.2% glutaraldehyde (Sigma-Aldrich, St. Louis, MO, USA) at room temperature for 1 h. The fixed cells were incubated at 37 °C in a CO_2-free environment for 12 h with a β-gal staining solution (1 mg/mL X-Gal, 40 mM citric acid-sodium phosphate buffer, 150 mM NaCl, 2 mM MgCl2, 5 mM potassium ferrocyanide, and 5 mM potassium ferricyanide; pH 6.0; Sigma-Aldrich). The stained (blue, positive) and unstained (negative) cells were counted by using phase contrast microscopy (Nikon, Tokyo, Japan) in five independent cultures.

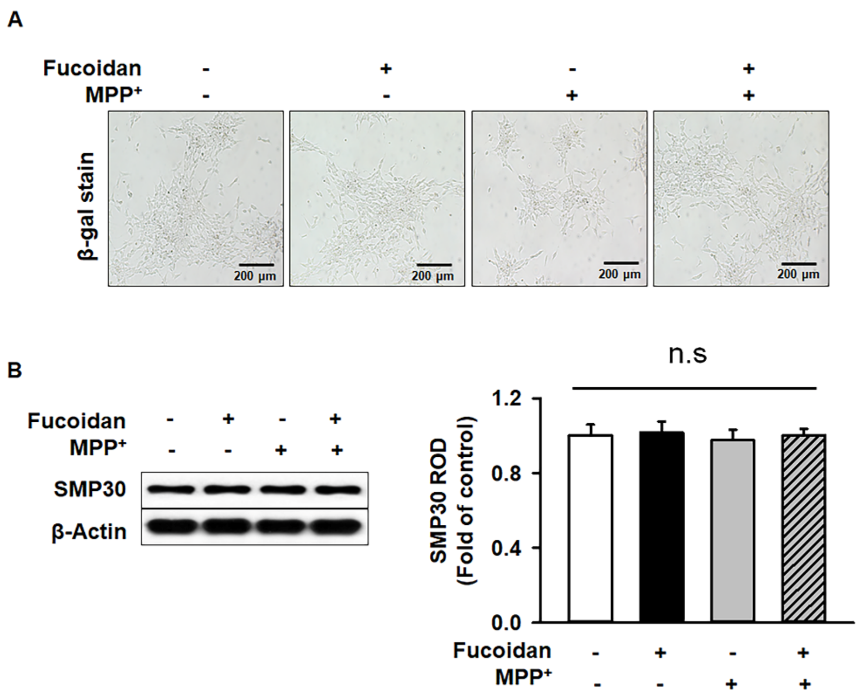

Supplemental Figure 1. Effect of fucoidan and MPP+ on senescence in SH-SY5Y cells. (A) Senescence-associated β-galactosidase (β-gal) staining in SH-SY5Y cells treated with fucoidan and/or MPP+. Scale bar = 200 μm. (B) Western blotting analysis for quantifying senescence marker protein 30 (SMP30) in SH-SY5Y cells treated with fucoidan and/or MPP+ (n = 3). The values represent the means ± SDs. n.s = no significance.

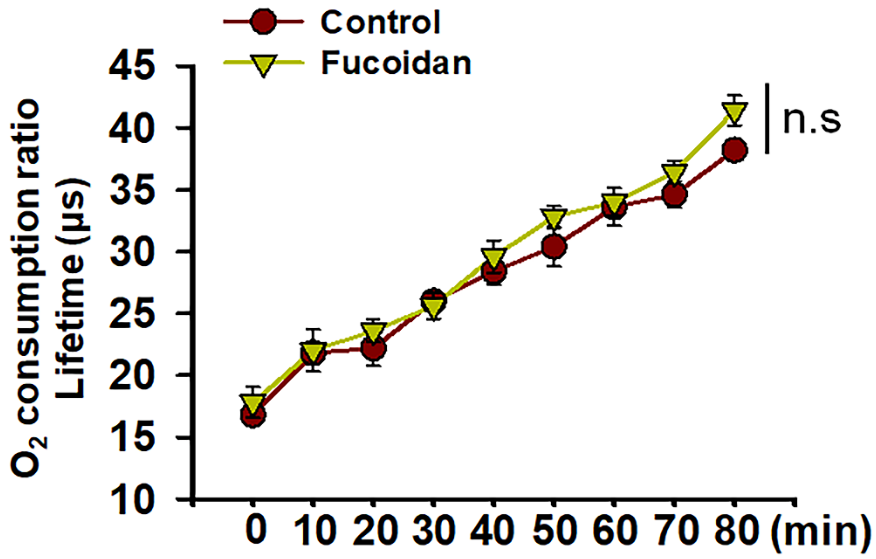

Supplemental Figure 2. Effect of fucoidan on mitochondrial oxygen consumption in SH-SY5Y cells. Mitochondrial oxygen consumption ratio in SH-SY5Y cells after treatment with fucoidan (50 μg/ml) for 24 h (n = 5). The values represent the means ± SDs. n.s = no significance.
